# Role of vitamin D_3_ in Treatment of Lumbar Disc Herniation—Pain and Sensory Aspects: Study Protocol for a Randomized Controlled Trial

**DOI:** 10.1186/1745-6215-15-373

**Published:** 2014-09-25

**Authors:** Mahsa Sedighi, Ali Haghnegahdar

**Affiliations:** Department of Neurosurgery, Shiraz Medical School, Shiraz University of Medical Sciences, PO Box 71345-1536, Shiraz, Iran; Department of Neurosurgery, Shiraz Medical School, Shiraz University of Medical Sciences, PO Box 71345-1536, Shiraz, Iran

**Keywords:** Inflammation, Lumbar disc herniation, Pain, Sensory, Vitamin D_3_

## Abstract

**Background:**

Vitamin D receptors have been identified in the spinal cord, nerve roots, dorsal root ganglia and glial cells, and its genetic polymorphism association with the development of lumbar disc degeneration and herniation has been documented. Metabolic effects of active vitamin D metabolites in the nucleus pulposus and annulus fibrosus cells have been studied. Lumbar disc herniation is a process that involves immune and inflammatory cells and processes that are targets for immune regulatory actions of vitamin D as a neurosteroid hormone. In addition to vitamin D’s immune modulatory properties, its receptors have been identified in skeletal muscles. It also affects sensory neurons to modulate pain. In this study, we aim to study the role of vitamin D_3_ in discogenic pain and related sensory deficits. Additionally, we will address how post-treatment 25-hydroxy vitamin D_3_ level influences pain and sensory deficits severity. The cut-off value for serum 25-hydroxy vitamin D_3_ that would be efficacious in improving pain and sensory deficits in lumbar disc herniation will also be studied.

**Methods/Design:**

We will conduct a randomized, placebo-controlled, double-blind clinical trial. Our study population will include 380 cases with one-level and unilateral lumbar disc herniation with duration of discogenic pain less than 8 weeks. Individuals who do not have any contraindications, will be divided into three groups based on serum 25-hydroxy vitamin D_3_ level, and each group will be randomized to receive either a single-dose 300,000-IU intramuscular injection of vitamin D_3_ or placebo. All patients will be under conservative treatment. Pre-treatment and post-treatment assessments will be performed with the McGill Pain Questionnaire and a visual analogue scale. For the 15-day duration of this study, questionnaires will be filled out during telephone interviews every 3 days (a total of five times). The initial and final interviews will be scheduled at our clinic. After 15 days, serum 25-hydroxy vitamin D_3_ levels will be measured for those who have received vitamin D_3_ (190 individuals).

**Trial registration:**

Iranian Registry for Clinical Trials ID: IRCT2014050317534N1 (trial registration: 5 June 2014)

## Background

Medical treatment is the first step in therapy for lumbar disc herniation (LDH), except for patients who require immediate surgical decompression. Drugs that are utilized in treatment of LDH pain and sensory deficits include muscle relaxants [1–3], analgesics [1, 2, 4–9], corticosteroids [1, 2, 10], antidepressants [4, 8, 11, 12] and antiepileptics [4, 8, 11–17].

Vitamin D is a secosteroid hormone that has many skeletal and nonskeletal functions [18–94]. In addition to its classic action on bone metabolism and osteoporosis [18, 19], its links and roles in relation to other diseases have been addressed in the literature (diabetes mellitus [18, 20–23], hypertension [24, 25], cardiovascular diseases [18, 26–29], multiple sclerosis [30–35], neurodegenerative diseases [36–39], neuropsychiatric diseases [39–44], inflammatory bowel disease [33, 45–49], dermatologic diseases [50–58], rheumatoid arthritis [47, 53, 59–61], systemic lupus erythematosus [60, 62–67], transplant rejection [68–70], cancer [18, 52, 68, 71–73], postherpetic neuralgia [74], corneal neuralgia [75], respiratory diseases [76–79], pregnancy complications [80–82], human reproductive issues [83–85], migraine headache [86], chronic low back pain [87, 88], chronic painful conditions and fibromyalgia [89, 90] and diabetic neuropathy [91–93]). Studies that have shed light on areas that have given us the scientific underpinning for our present proposal are described below.

1. Vitamin D has been called a neurosteroid hormone [39, 74, 94–109], given its protective role against neurotoxicity and detoxification pathways [74, 94, 96–108] and also its receptors in different parts of the central nervous system [36, 94–96, 106–114].
2. Vitamin D receptors are present in the spinal cord, nerve roots, dorsal root ganglia and glial cells [94, 96, 97, 113, 115–118].
3. Vitamin D receptor gene polymorphism has a role in the development of lumbar disc degeneration and herniation [119–123].
4. Discs are composed largely of avascular tissue with a great sensitivity to its nutritional supply and excretion of waste products, and the balance between these two processes is an important factor that could lead to disc degeneration [124–127]. The effects of active vitamin D metabolites in nucleus pulposus and annulus fibrosus cells have been studied [128]. Vitamin D inhibits and decreases production of monocyte chemoattractant protein 1, thrombopoietin, vascular endothelial growth factor and angiogenin by human annulus cells *in vitro* [129]. As mentioned above, vitamin D affects detoxification pathways which are of importance in disc cell nutritional balance.
5. Vitamin D possesses immune regulatory properties which can downregulate proinflammatory cytokines and upregulate anti-inflammatory cytokines [22, 32, 36, 46–48, 58, 67, 70, 74, 78, 90, 94, 96, 130–146].
6. Vitamin D has properties that defend against cell injury caused via free radicals, reactive oxygen species, glutathione and glutamate [74, 94, 96–108, 136, 147–149].
7. Vitamin D has a role in pain by downregulating inflammatory cytokines that produce pain (a) directly, (b) by stimulating release of pain mediators, (c) by upregulating anti-inflammatory cytokines to help the body combat inflammation, (d) by its role in eliminating toxic metabolites or (e) by increasing the antioxidant pool. It also affects sensory neurons to modulate pain [114], influences neuron excitability [96] and acts at the level of substantia gelatinosa and spinal ganglion in the process of sensory perception [118]. In addition, its status affects pain sensitivity and opiate activity [150].
8. The role of the vitamin D receptor in skeletal muscles [151–155] and its effects on muscle strength and function have been identified [156–159].

In addition to the information described above, many studies about changes that occur in LDH have been done, as outlined below.

1. The contribution of inflammatory cytokines in the pathogenesis of LDH has been widely addressed in the literature. The herniated nucleus pulposus, either with immunogenic properties itself or by inducing an immunologic response in the nerve roots, dorsal root ganglia and surrounding muscles, is the starting point for the cascade of inflammation initiated through immune cell activation and infiltration and cytokine release [160–184].
2. Neuropathic pain involves the activation of neurons, glial cells and the immune system [185, 186]. Dorsal root ganglia and dorsal roots play important roles in LDH, not only by the effect of released inflammatory cytokines but also by actively amplifying inflammation by producing proinflammatory cytokines and pain mediators that affect pain perception and nociception. Among these substances is brain-derived neurotrophic factor. Its receptor has been identified in intervertebral discs, with its expression being increased during inflammatory conditions such as LDH and its neuroimmunomodulatory role in the dorsal root of the spinal cord [185, 187–204]. The other factor is glial cell–derived neurotrophic factor (GDNF). It has been shown that GDNF reduces neuropathic pain states [188, 190, 205–208]. Interestingly, vitamin D affects neuropathic pain by directly suppressing inducible nitric oxide that is expressed in glial cells [96, 136] or by affecting other substances, such as reactive oxygen species or glutamate. Given the immunomodulatory action of vitamin D, it is possible that it could downregulate inflammatory chemokines released by glial cells [96, 185–189, 209–215]. It has been suggested that vitamin D attenuates ischemia-induced brain injury that is thought to be mediated through upregulation of GDNF, in addition to its role in nitric oxide (NO) suppression [216]. The results of other studies support the hypothesis that GDNF is upregulated by vitamin D [90, 94, 96, 190, 217]. Interleukin 6 (IL-6) and tumor necrosis factor α produced by glial cells were shown to be downregulated by vitamin D [94, 96, 136], as were glial cell release of NO [188, 218, 219], prostaglandin [188], IL-1 and IL-6 [218], which, as described below, could be suppressed by vitamin D administration. Glial cells have glutamate receptors that are important in the process of nociception [220–224]. Therefore, vitamin D, through its immunoregulatory properties, affects another important cell population that is inflamed in disc herniation, either through suppressing neurotoxic agents or by its action on neurotrophins.

Some specific inflammatory cytokines and pain mediators that are involved in LDH and vitamin D immunomodulatory effects with regard to these specific substances are described in Table 1.

**Table 1:** **Vitamin D effects on substances involved in lumbar disc herniation**

Vitamin D actions [references]	LDH [references]
IFN-γ: D [46, 65, 72, 88, 94, 144]	E [160, 171, 179, 180]
IL-1: D [46, 65, 72]	E [173, 225–227]
IL-2: D [46, 65, 72, 88, 92, 94, 139]	
IL-4: D [46]	E [179]
IL-5: D [67]	
IL-6: D [32, 46, 72, 92, 94, 136, 141]	E [165, 176, 181, 228–230]
IL-8	E [164, 225, 231]
IL-10: U [32, 47, 67, 74, 90, 94, 96, 144, 226, 227]	
IL-12: D [22, 32, 67, 139, 140]	E [181, 182]
IL-17: D [47, 90]	E [181]
MCP: I [129]	E [164, 175]
MMP: I [232–240]	E [176, 190, 228, 241–243]
ROS: I [98, 101, 102, 106, 238]	E [244]
NO: I [245]	E [126, 148, 176, 190, 228, 246–249]
Glutamate: I [101, 147]	E [220, 221]
Glutathione: I [96, 106, 148]	
PG: I [250]	E [176, 190, 228, 243, 251, 252]

D, Downregulation; E, Expression; I, Inhibition; IFN-γ, Interferon γ; IL, Interleukin; LDH, Lumbar disc herniation; MCP, Monocyte chemoattractant protein; MMP, Matrix metalloproteinase; NO, Nitric oxide; PG, Prostaglandin; ROS, Reactive oxygen species; U, Upregulation.

3. Detailed study of inflammatory cytokines and subsequent pain mediators released in LDH has shown that there is a shift toward type 1 T-helper cell activity [164, 177, 181, 182, 228].
4. Vitamin D decreases the number and function of type 1 T-helper cells [47, 48, 67, 90, 253].
5. Muscle changes associated with low back pain have been studied [254–258]. Studies have shown how muscles are affected by LDH [259–266]. Atrophy of type II muscle fibers [259–261, 263] or atrophy of both types I and II muscle fibers [260] and adipocyte enlargement are examples of how muscles are targeted by LDH [264]. Vitamin D deficiency–associated histochemical changes in muscles somehow resemble those seen in LDH-affected muscles with atrophy of type II muscle fibers [267–271] and enlarged interfibrillar spaces and fat infiltration and glycogen granules [271–274]. Another interesting aspect of vitamin D deficiency is how it promotes skeletal muscle hypersensitivity and sensory hyperinnervation [275]. Vitamin D supplementation was shown to increase the diameter of type II muscle fibers [181, 276]. It also influences transdifferentiation of muscle cells to adipose cells [277]. With regard to the presence of vitamin D receptor in skeletal muscles [151–155], its effect on muscle growth and proliferation [278–282] and the changes seen in muscles after LDH, we propose that vitamin D supplementation also influences muscle changes in this condition.

## Methods/Design

### Design of the study

We will conduct a randomized, placebo-controlled, double-blind clinical trial.

### Statement of ethical approval

This study was approved by the local research ethics committee of Shiraz University of Medical Sciences, Shiraz, Iran (CT-P-92-6632).

## Informed consent

Informed consent will be obtained from all participants.

### Setting

We will recruit patients who have appointments at the neurosurgery outpatient departments of the university-affiliated hospitals of Shiraz, Iran.

### Participants

We will recruit 380 patients with LDH proven by physical examination and confirmed by magnetic resonance imaging.

### Intervention

Patients in the intervention arm will receive single-dose intramuscular injections of 300,000 IU of vitamin D_3_ (1 ml). Individuals will be informed about the nature of this study.

#### Inclusion criteria

The following are the inclusion criteria:

1. Single-level LDH
2. No coexistent or preexisting spine pathology (for example, spondylolysis, spondylolisthesis, infection, tumors, fracture)
3. Discogenic pain duration less than 8 weeks from onset to physician’s evaluation
4. Compliance with the study protocol
5. Normal laboratory studies that do not contraindicate vitamin D_3_ injection

#### Exclusion criteria

The following are the exclusion criteria:

1. Daily supplementation of more than 800 IU of vitamin D_3_
2. Serum calcium level above 10.5 mg/dl
3. Hypercalciuria (spot urine calcium/creatinine ratio above 0.4)
4. Lymphoma, sarcoidosis, tuberculosis (TB), hyperparathyroidism, celiac disease or malabsorption syndromes
5. History of kidney stones
6. History of inflammatory back pain
7. Impaired renal function tests (glomerular filtration rate less than 30 ml/min/1.73 m^2^)
8. Impaired hepatic function tests
9. Abnormal serum phosphorus, alkaline phosphatase and parathyroid hormone values
10. Fasting blood sugar above 126 mg/dl
11. Previous spine surgery
12. History of trauma
13. Taking anticonvulsant, anti-TB medications or vitamin D_3_ analogues
14. Cauda equine syndrome that requires emergency surgical decompression

#### Laboratory Assessments

The following laboratory workups will be performed for all included participants: serum 25-hydroxy vitamin D_3_ level, serum calcium, serum phosphorus, alkaline phosphatase, parathyroid hormone, liver function tests (bilirubin (direct and total), alanine transaminase, aspartate transaminase, total protein, total albumin), blood urea nitrogen, creatinine, spot urine for calcium and fasting blood sugar. Clinic-based pre-intervention interviews and physical examinations will include the following:

1. McGill Pain Questionnaire: The McGill Pain Questionnaire is used to evaluate different pain qualities and intensities. This questionnaire consists of four major descriptors: sensory, affective, evaluative and miscellaneous. Each descriptor has its own rank value. The sum of these rank values is the pain rating index. Present pain intensity is measured on scale from 0 to 5 [281].
2. Visual analogue scale (VAS) to evaluate low back pain and radicular pain: A VAS is a pain measurement scale that incorporates numbers and faces to depict the severity of pain. It is usually a 100-mm line. Its ends show the pain extremes [229, 282].
3. A physical examination to detect any sensory deficits.

### Randomization

Patients will be categorized on the basis of their serum 25-hydroxy vitamin D_3_ levels into three groups:

-  Group 1: Optimum 25-hydroxy vitamin D_3_ level (32 to 50 ng/ml)
-  Group 2: Deficient 25-hydroxy vitamin D_3_ level (less than 10 ng/ml)
-  Group 3: Insufficient 25-hydroxy vitamin D_3_ level (less than 32 ng/ml)

Each of the groups will be randomized, based on randomly computer-generated numbers, into two groups to receive intramuscular injection of either 300,000 IU of vitamin D_3_ (1 ml) or distilled water (1 ml). All patients will be prescribed daily 15 mg Meloxicam capsules. Our study population will be warned verbally and in writing about the potential for severe adverse side effects of vitamin D_3_ (nausea, vomiting, abdominal pain, metallic taste, breathing difficulties). They will have access to emergency department care should side effects occur.

The study will last 15 days. After vitamin D_3_ injection, patients will be contacted by telephone every 3 days to assess the sensory and pain effects of vitamin D_3_ with the McGill Pain Questionnaire and the VAS (a total of five times). Participants will be provided with the VAS so that they can look at the scale and report their pain severity during the telephone interviews.

The following are the final post-treatment evaluations that will be carried out at the clinic:

1. McGill Pain Questionnaire
2. VAS (for low back pain and radicular pain)
3. Physical examination to detect any sensory deficits

Post-treatment 25-hydroxy vitamin D_3_ levels (after 15 days) will be measured for those participants who have received vitamin D_3_ (*N* = 190).

### Statistical analysis

Data will be assessed by analysis of variance and paired tests.

## Discussion

On the basis of the inflammatory nature of disc herniation and the immunomodulatory effects of vitamin D, as well as the existence of vitamin D receptors in various parts of areas that are affected in the process of disc herniation, we propose a novel role for vitamin D in the treatment of discogenic pain and sensory deficits related to this pathology. We hypothesized that vitamin D_3_ plays a role in reducing the severity of discogenic pain and that vitamin D_3_ can improve discogenic-related sensory deficits.

The following are our general objectives in this trial:

1. Effect of vitamin D_3_ on discogenic pain
2. Effect of vitamin D_3_ on discogenic sensory deficits
3. Effect of posttreatment 25-hydroxy vitamin D_3_ level on pain and sensory deficit severity
4. Determining a cut-off level of 25-hydroxy vitamin D_3_ that is efficient in improving pain and sensory deficits

The following are our applicative objectives:

1. Proposing vitamin D_3_ as part of medical treatment for LDH
2. Improving LDH patients’ quality of life
3. Decreasing the economic and health burden of LDH

Our ultimate goal in this study is to introduce a new treatment strategy for the treatment of discogenic pain.

## Trial status

The study protocol has been approved by the Vice-Chancellor for Research of Shiraz University for Medical Sciences. Recruitment has not been initiated.

## Abbreviations

ALT: Alanine transaminase
AST: Aspartate transaminase
D: Downregulation
E: Expression
I: Inhibition
IL: Interleukin
IFN-γ: Interferon γ
LDH: Lumbar disc herniation
MCP: Monocyte chemoattractant protein
MMP: Matrix metalloproteinase
NO: Nitric oxide
PG: Prostaglandin
ROS: Reactive oxygen species
U: Upregulation.
